# Development of cochlear spiral ligament fibrocytes of the common marmoset, a nonhuman model animal

**DOI:** 10.1038/s41598-023-39003-x

**Published:** 2023-07-21

**Authors:** Makoto Hosoya, Kaho Iwabu, Tsubasa Kitama, Takanori Nishiyama, Naoki Oishi, Hideyuki Okano, Hiroyuki Ozawa

**Affiliations:** 1 Department of Otolaryngology, Head and Neck Surgery, Keio University School of Medicine, 35 Shinanomachi Shinjuku-ku, Tokyo, 160-8582 Japan; 2 Department of Physiology, Keio University School of Medicine, 35 Shinanomachi Shinjuku-ku, Tokyo, 160-8582 Japan; 3 Laboratory for Marmoset Neural Architecture, Center for Brain Science, RIKEN, 2-1 Hirosawa Wako, Saitama, 351-0193 Japan

**Keywords:** Developmental biology, Neuroscience

## Abstract

Spiral ligament fibrocytes generate potassium gradients, which hair cells require to convert mechanical sound waves into electrical palsy. Together with the stria vascularis, they regulate endolymph electrolyte homeostasis. Developing spiral ligament fibrocytes and generating endocochlear potential with an appropriate endolymph ion composition are essential for hearing. Understanding spiral ligament fibrocyte development is useful for studying age-related and genetic hearing loss, as well as for regenerative therapy and cochlear immunology. Despite interspecies differences, most studies of cochlear development have been conducted in rodent models due to the difficulty of using human fetal samples. This study investigated the cochlear development of spiral ligament fibrocytes in a small New World monkey species, the common marmoset (*Callithrix jacchus*). We examined the developmental expression of specific genes in spiral ligament fibrocytes, including those essential for the generation of endolymphatic potential. Our results showed that this animal model of spiral ligament fibrocyte development is similar to that of humans and is a suitable alternative for the analysis of human cochlear development. The time course established in this study will be useful for studying the primate-specific developmental biology of the inner ear, which may lead to novel treatment strategies for human hearing loss.

## Introduction

The inner ear is a peripheral sensory organ essential for hearing and balance. The cochlea, part of the inner ear, is mammals' peripheral organ responsible for hearing. In the cochlea, hair cells convert the mechanical sensation of sound into neuroelectric pulses. These pulses eventually reach the auditory cortex of the brain, where neurons can perceive the neuroelectric stimuli converted from sound waves. The generation of this intrinsic electrical impulse in the hair cells is driven by a concentration gradient of potassium ions between the hair cells and the endolymph facing the hair cells.

The spiral ligament fibrocytes of the cochlea are the major tissue that generates the concentration gradient of potassium ions essential for hearing. Spiral ligament fibrocytes also regulate the composition of endolymph in collaboration with the stria vascularis^1,2^. Therefore, the finely-tuned development of spiral ligament fibrocytes in the cochlea is essential for normal hearing^3^. These spiral ligament fibrocytes are composed of five cell types according to their location in the spiral ligament: type-IV fibrocytes. Depending on the type, these spiral ligament fibrocytes express specific transporters, channels, or gap junctions. They collaborate to maintain the electrolyte ion homeostasis in the cochlea. In addition, these spiral ganglion fibrocytes play important physiological roles in the cochlear immune response, inflammation, aging, regulation of cochlear blood flow, and glutamate homeostasis^4,5^.

Previous studies have mainly focused on cochlear development in rodent models. However, human cochlear development is poorly understood because it occurs in late gestation^6–8^, and ethical issues preclude the study of late-term fetal samples. Furthermore, species differences in cochlear development between rodents and humans have been reported, and findings on cochlear development in rodent models cannot necessarily be directly extrapolated to humans^6^. To overcome this sampling limitation and minimize species differences, the small primate model of the common marmoset (*Callithrix jacchus*) has been used to understand cochlear development^9–12^ and genetic or age-related hearing loss^13^. Similar approaches have been used to study the central nervous system^14^. We have previously reported on the basic anatomical stages of cochlear development in the common marmoset compared to humans and mice, including the expression patterns in hair cells, supporting cells, spiral ganglion neurons, stria vascularis, and other conventional markers of cochlear cells^9–12^. In addition, we identified several differences between rodents and marmosets in the development of hair cells, spiral ganglion neurons, and stria vascularis. Moreover, we demonstrated similarities to the human developmental process^9,10,12^.

Research on the development of spiral ligament fibrocytes in non-human primates is still in its infancy. Such studies would reveal interspecies differences in the development of spiral ligament fibrocytes. In addition, knowledge of primate development will be useful in regenerative studies of human spiral ligament fibrocytes.

In this study, we present a detailed description of the cochlear development in *C. jacchus*. Furthermore, as in humans, cochlear development in primates is slower than in rodents^6,9^. As precisely demonstrated in hair cells, spiral ganglion neurons, and stria vascularis, it is believed that this animal model is better suited to detect short or transient developmental changes in gene expression patterns.

## Materials and methods

### Specimens

Cadaverous temporal bone samples from common marmosets at E96 (n = 3), E109 (n = 3), E115 (n = 3), E120 (n = 3), and P0 (n = 3) were used in this study, as same as our previous reports^9,12^. Adult animals were anesthetized with isoflurane inhalation (1.5–4%) and Caesarion section was performed. Embryos were anesthetized on ice deeply. The animal experiments were approved by the Animal Experiment Committee of Keio University (Approval number: 11006, 08020) and performed according to the guidelines of the National Institutes of Health and the Ministry of Education, Culture, Sports, Science, and Technology of Japan.

### Tissue preparation

Temporal bones were dissected immediately after euthanasia and fixed in 4% paraformaldehyde in phosphate-buffered saline (PBS) overnight. Specimens were embedded in Tissue-Tek O.C.T. compound (Sakura Finetechnical Co., Ltd., Tokyo, Japan) for cross-sectioning. P0 specimens were decalcified in Decalcifying Solution B (Wako, Osaka, Japan) for 1 week and then embedded in Tissue-Tek O.C.T. compound (Sakura Finetechnical Co., Ltd., Tokyo, Japan) for cross-sectioning, as same as our previous reports^9,12^. Seven-micrometer sections were used for immunohistochemical analysis.

### Antibodies

The following primary antibodies were used:

Anti-CALD1 (mouse IgG1, MS-1251-P0, NeoMarkers, Fremont, CA, USA, 1:500), anti-COCH (rabbit IgG, HPA050122, Atlas Antibodies, Bromma, Sweden, 1:200), anti-CCN2 (goat IgG, sc14939, Santa Cruz Biotechnology, Santa Cruz, CA, USA, 1:200), anti-ATP1B1 (mouse IgG2a, ab2873, Abcam, Cambridge, UK, 1:1000), anti-ATP1A1 (rabbit IgG, ab76020, Abcam, Cambridge, UK, 1:1000), anti-SLC12A2 (goat IgG, sc-21545, Santa Cruz Biotechnology, Santa Cruz, CA, USA, 1:1000), anti-KCNJ16 (rabbit IgG, APC123, Alomone labs, Jerusalem, Israel, 1:1000), anti-CA2 (rabbit IgG, sc-25596, Santa Cruz Biotechnology, Santa Cruz, CA, USA, 1:500), anti-GLUT1 (rabbit IgG, ab115730, Abcam, Cambridge, UK, 1:500), anti-COL2A1 (mouse IgG2b, sc-52658, Santa Cruz Biotechnology, Santa Cruz, CA, USA, 1:100).

The following secondary antibodies were used: donkey anti-mouse IgG, Alexa Fluor Plus 488 (A32766, Invitrogen, Waltham, MA, USA, 1:500); donkey anti-rabbit IgG, Alexa Fluor Plus 555 (A32794, Invitrogen, Waltham, MA, USA, 1:500); and donkey anti-goat Alexa Fluor Plus 647 (A32849, Invitrogen, Waltham, MA, USA, 1:500).

### Immunohistochemistry

After a brief wash with PBS, the sections were heated (80 °C) in 10 µM citrate buffer (pH 6) for 15 min. After another brief wash, the sections were pre-blocked in PBS containing 10% normal serum for 1 h at room temperature, incubated with the relevant primary antibodies overnight at 4 °C, and then incubated with Alexa Fluor-conjugated secondary antibodies for 60 min at room temperature. The nuclei were counterstained with Hoechst 33258.

### Ethical approval and consent to participate

The animal experiments were approved by the Animal Experiment Committee of Keio University (approval number: 11006, 08020) and were performed in accordance with ARRIVE guidelines and the guidelines of the National Institutes of Health and the Ministry of Education, Culture, Sports, Science, and Technology of Japan.

## Results

### Developmental expression patterns of *CALD1*, *COCH* and *CCN2*

**Figure 1:**
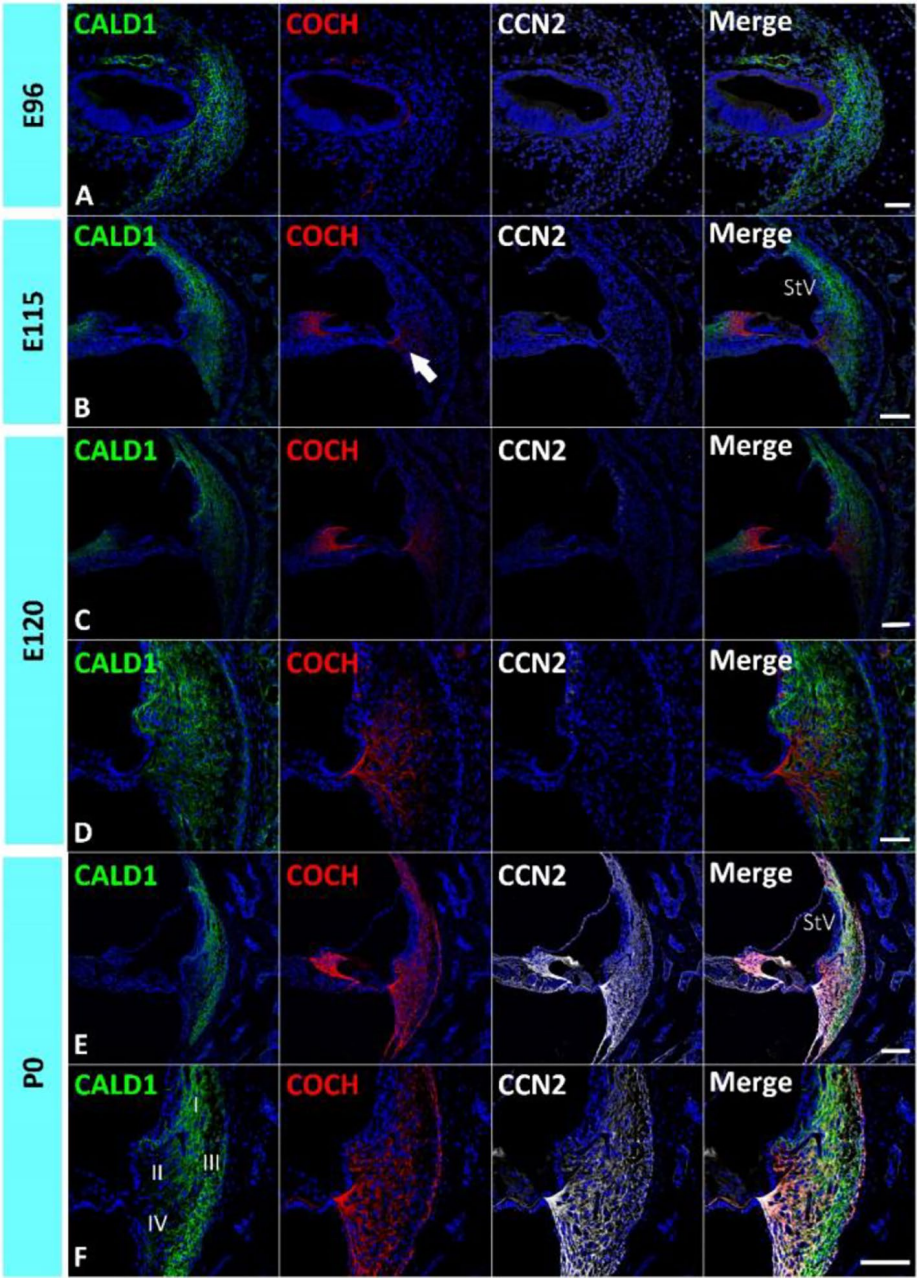
Expression of *CALD1*, *COCH*, and *CCN2*. (**A**) Expression of *CALD1, COCH,* and *CCN2* in the E96 cochlea. Among them, only *CALD1* expression was observed in the developing periotic mesenchymal tissue. (**B**) Expression of *CALD1, COCH,* and *CCN2* in the E115 cochlea. While *CALD1* expression was widely observed in the spiral ligament fibrocytes, only weak *COCH* expression was detected at this stage (arrow in **B**). No *CCN2* expression was observed in spiral ligament fibrocytes at this stage. (**C**,**D**) Expression of *CALD1, COCH,* and *CCN2* in the E120 cochlea. At this stage, *CALD1* expression is widely observed in spiral ligament fibrocytes, and a relatively broader expression of *COCH* was detected compared with E115. However, *CCN2* expression could not be detected at E120. (**E**,**F**) Expression of *CALD1*, *COCH,* and *CCN2* in the P0 cochlea. At P0, both *COCH* and *CCN2* expressions were observed ubiquitously in spiral ligament fibrocytes. *CALD1* expression in type II, IV, and V fibrocytes was reduced and restricted to type I and III at this stage. Nuclei were counterstained with Hoechst (blue). Scale bar 50 µm in (**A**,**D**), 100 µm in (**B**,**C**,**E**,**F**). *StV* stria vascularis, *I* type I fibrocytes, *II* type II fibrocytes, *III* type III fibrocytes, *IV* type IV fibrocytes. (**A**–**F**) Basal turns.

The common marmoset has a gestation period of approximately 150 days^15^. Previously, we reported that E115 of the marmoset is equivalent to P9 of the mouse and 20 weeks of gestation in humans for cochlear development, and P0 of the marmoset is equivalent to P14 of the mouse and 24 weeks of gestation weeks in humans^9^. First, this study examined the expression patterns of *CALD1*, *COCH*, and *CCN2* (Fig. 1), which have been reported to be highly expressed in spiral ligament fibrocytes of the adult marmoset cochlea^16^.

*CALD1* encodes Caldesmon, a calmodulin-binding protein. Caldesmon tonically inhibits the ATPase activity of myosin in smooth muscle. Caldesmon is expressed in type I and III fibrocytes in the spiral ligament fibrocytes of the mature cochlea in both rodents and primates^16,17^. *CALD1* expression was previously detected in the developing cochlea of the common marmoset as early as E96 (Fig. 1A)^9^. Therefore, we used this protein as a conventional marker for spiral ligament fibrocytes. Its expression was observed in the developing periotic mesenchymal tissue, but not in the sensory epithelium. Until E120, *CALD1* expression was widely observed in spiral ligament fibrocytes (Fig. 1B–D). However, at P0, *CALD1* expression was restricted to type I and III fibrocytes (Fig. 1E).

*COCH* encodes Cochlin, an extracellular matrix (ECM) protein that is highly abundant in the cochlea and vestibule of the inner ear and is the major non-collagen component of the ECM of the inner ear^18^. *COCH* has also been identified as the causative gene for DFNA9, a hereditary hearing loss. Cochlin is also used clinically as a diagnostic marker for perilymphatic fistula^19^. In the developing cochlea of the common marmoset, a low level of *COCH* expression was detected at E115, whereas no expression was observed in the E96 cochlea (Fig. 1A,B). At E120, *COCH* expression was observed in presumptive type II and IV fibrocytes (Fig. 1C,D). At P0, *COCH* expression was widely observed in spiral ligament fibrocytes, as previously reported in the adult primate cochlea^16^ (Fig. 1E,F).

*CCN2* encodes connective tissue growth factor (CTGF), a matricellular protein belonging to the CCN family of ECM-associated heparin-binding proteins^20,21^. *CCN2* expression has been previously reported in spiral ligament fibrocytes of rodents^22–24^, and the common marmoset^16^. In the developing cochlea of the common marmoset, no *CCN2* expression was detected until E120 (Fig. 1C,D). At P0, ubiquitous expression of *CCN2* was observed in spiral ligament fibrocytes (Fig. 1E,F).

### Developmental expression patterns of *ATP1A1*, *ATP1B1*, and *SLC12A2*

**Figure 2:**
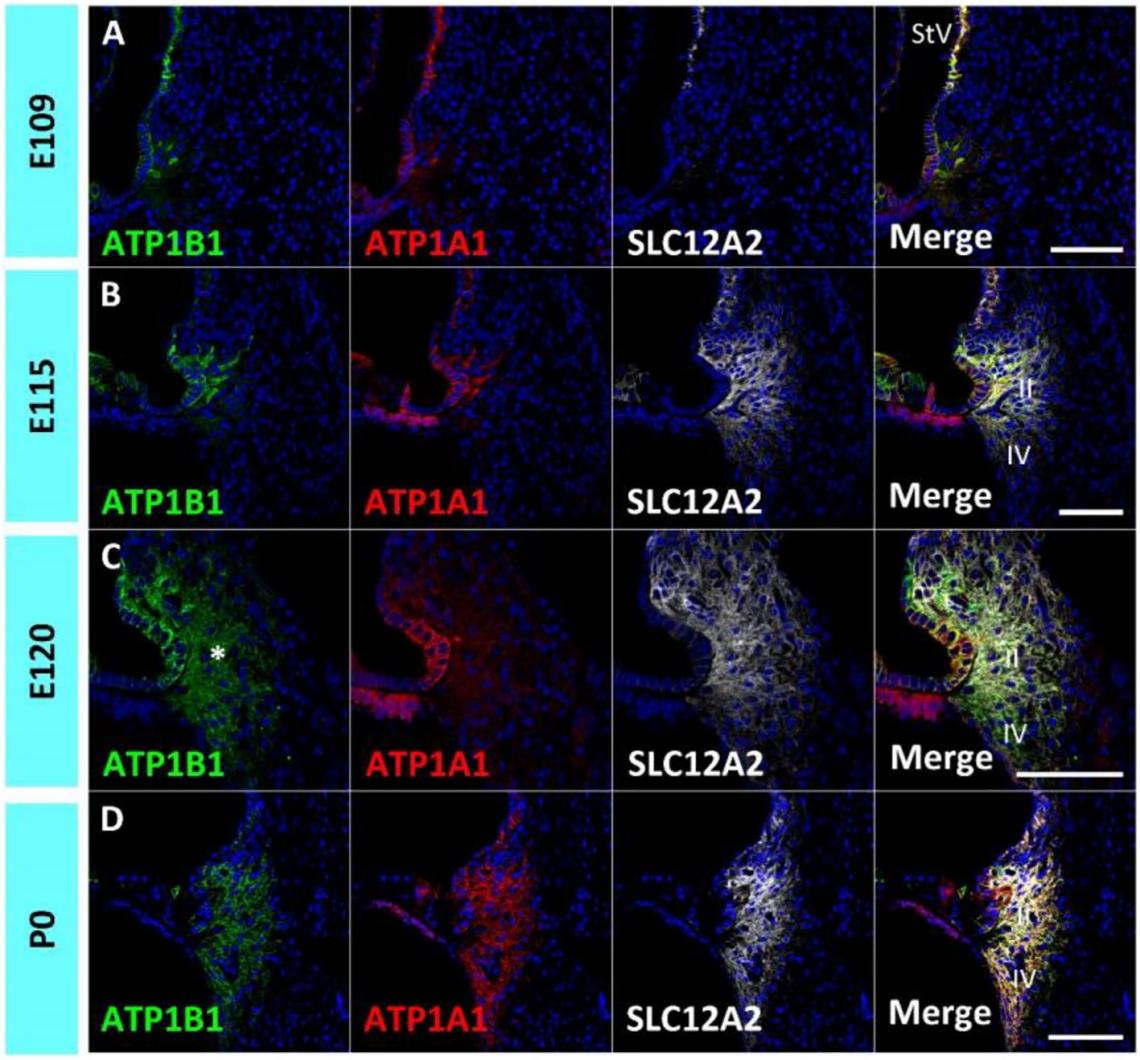
Expression of ATP1B1, ATP1A1, and SLC12A2. (**A**) Expression of *ATP1B1, ATP1A1,* and *SLC12A2* in the E109 cochlea. No expression of *ATP1B1, ATP1A1,* and *SLC12A2* expressions was detected in spiral ligament fibrocytes. (**B**) Expression of *ATP1B1, ATP1A1,* and *SLC12A2* in E115 cochlea. While *SLC12A2* expression could not be observed in the spiral ligament fibrocytes, no *ATP1B1* and *ATP1B1* expression could be detected at this stage. (**C**) Expression of *ATP1B1, ATP1A1*, and *SLC12A2* in E120 cochlea. At this stage, *ATP1B1* expression is observed in type II and IV spiral ligament fibrocytes (asterisk in **C**). In contrast, no *ATP1A1* expression was detected at this stage. (**D**) Expression of *ATP1B1, ATP1A1,* and *SLC12A2* in the E120 cochlea. At P0, *ATP1B1, ATP1A1,* and *SLC12A2* expression was observed in type II and IV fibrocytes. The nuclei were counterstained with Hoechst (blue). Scale bar 100 µm, *StV* stria vascularis, *II* type II fibrocytes, *IV* type IV fibrocytes. (**A**–**D**) Basl turns.

Homeostatic regulation of sodium and potassium in the endolymph, in conjunction with the stria vascularis, is one of the most important functions of spiral ligament fibrocytes. The expression of sodium and potassium transporters has been reported in the developing rodent cochlea^25–27^, and their expression during development is essential for normal hearing acquisition^28,29^. Therefore, this study examined the expression of *ATP1A1*, *ATP1B1*, and *SLC12A2*, which have been reported to be expressed in the developing stria vascularis^12^ and spiral ligament fibrocytes of the adult marmoset cochlea^16^ (Fig. 2).

*ATP1A1* and *ATP1B1* encode the Na^+^/K^+^-transporting ATPase subunits alpha-1 and beta-1^30,31^ that are responsible for establishing and maintaining the electrochemical gradients of Na^+^ and K^+^ ions across the plasma membrane. The cooperative activities of proteins in the basal membrane of marginal cells in the stria vascularis are essential for K^+^ cycling and the formation of the endocochlear potential in the lateral wall^32^. In developing marmoset spiral ligament fibrocytes, neither *ATP1A1* nor *ATP1B1* expression could be detected by E115, whereas both were detected in the stria vascularis (Fig. 2A,B). *ATP1B1* expression was detected in spiral ligament type II and IV fibrocytes of the cochlea at E120, but no *ATP1A1* expression was detected at this stage (Fig. 2C). At P0, both *ATP1A1* and *ATP1B1* expression was detected in spiral ligament type II and IV fibrocytes (Fig. 2D).

*SLC12A2* (Solute Carrier Family 12 Member 2) encodes the Na^+^/K^+^/2Cl^−^ cotransporter (NKCC1), which is essential for normal hearing^29,33^. Developmental expression patterns of the rodent cochlea have been reported^25^. In the spiral ligament fibrocytes of the developing cochlea of the common marmoset, no expression was observed until E109 (Fig. 2A). At E115, *SLC12A2* expression was observed in type II fibrocytes, and a weak expression was detected in type IV fibrocytes (Fig. 2B). At E120 and P0 cochlea, SLC12A2 expression was observed in type II and IV fibrocytes, similar to that in the adult marmoset cochlea (Fig. 2C,D).

### Developmental expression patterns of *KCNJ16*

**Figure 3:**
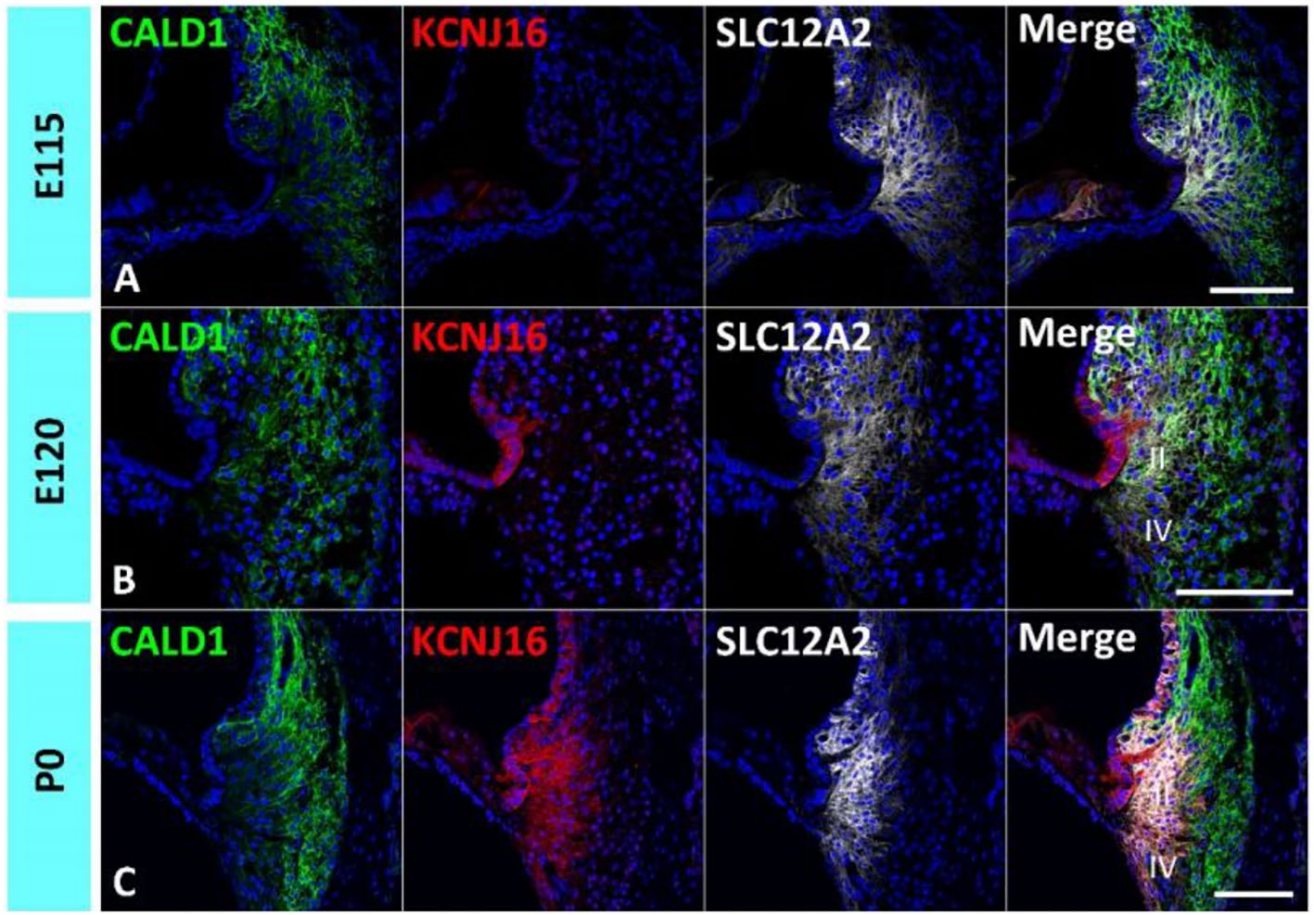
Expression of *KCNJ16*. (**A**) Expression of *KCNJ16* in the E115 cochlea. No *KCNJ16* expression could be detected in the spiral ligament fibrocytes. (**B**) Expression of *KCNJ16* in the E120 cochlea. No expression of *KCNJ16* could still be detected in the spiral ligament fibrocytes, while *KCNJ16* expression could be detected in outer sulcus cells at this stage. (**C**) Expression of *KCNJ16* in the P0 cochlea. *KCNJ16* expression could be detected in type II and IV spiral ligament fibrocytes at this stage. The nuclei were counterstained with Hoechst (blue). Scale bar 100 µm, *II* type II fibrocytes, *IV* type IV fibrocytes. (**A**–**C**) Basal turns.

Next, we investigated another potassium recycling-related gene in cochlear spiral ligament fibrocytes; *KCNJ16* (Fig. 3). *KCNJ16* encodes the Kir 5.1 protein, an integral membrane protein, and an inward-rectifier-type potassium channel. Kir5.1 expression has been reported in type II, IV, and V spiral ligament fibrocytes in rodents^34^. While defects in *KCNJ16* gene cause sensorineural hearing loss in human patients^35^; however, in mice, the *KCNJ16* gene is not essential for auditory function^36^. In the developing cochlea of the common marmoset, no *KCNJ16* expression was observed in the spiral ligament fibrocytes at E120, while *KCNJ16* expression in the root cells was detected at E120 (Fig. 3A,B). In the P0 cochlea, *KCNJ16* expression was observed in type II and IV spiral ligament fibrocytes (Fig. 3C).

### Developmental expression patterns of *CA2* and *SLC2A1*

**Figure 4:**
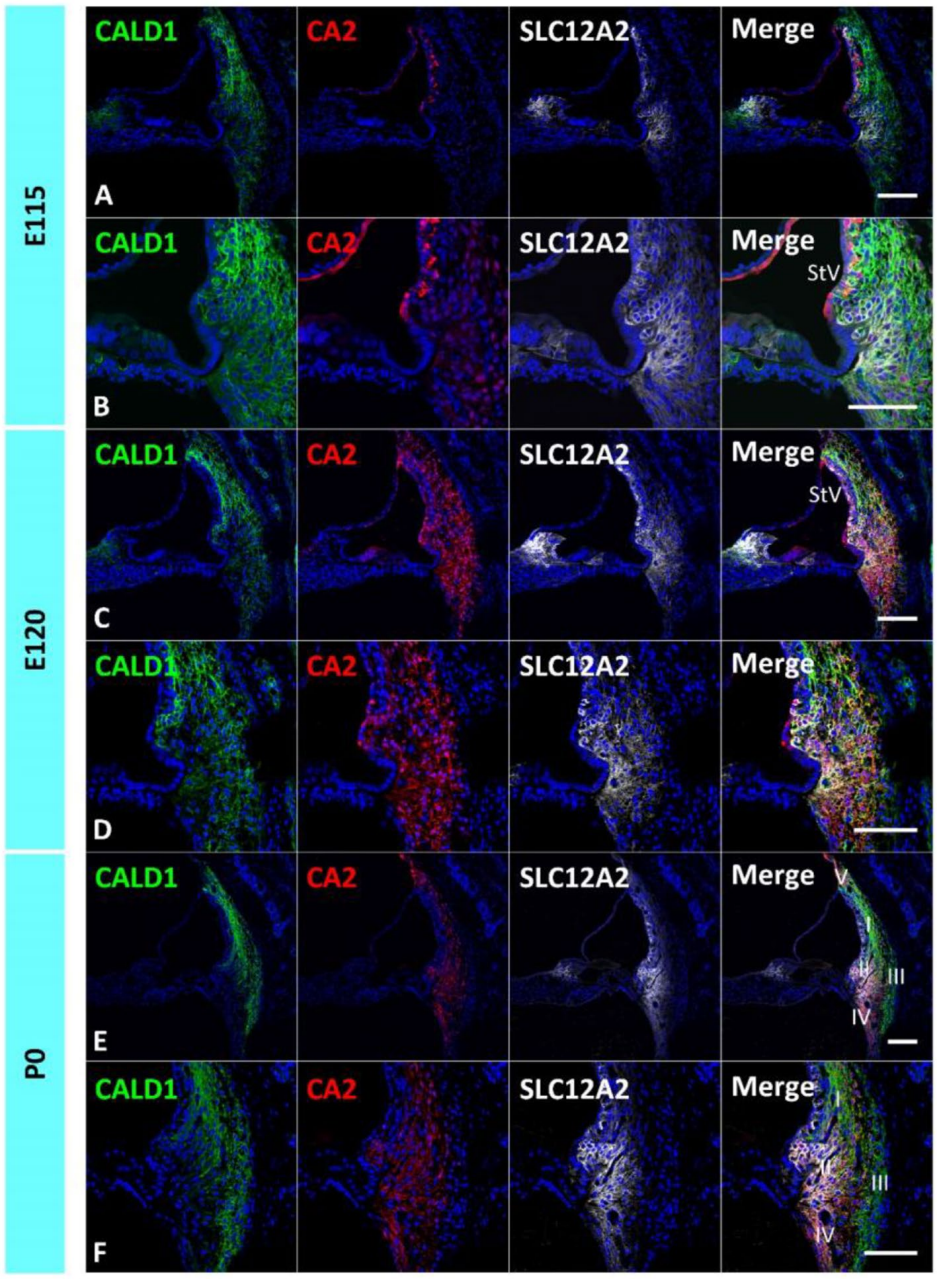
Expression of *CA2*. (**A**,**B**) Expression of *CA2* in the E115 cochlea. No *CA2* expression could be detected in the spiral ligament fibrocytes, while its expression in the stria vascularis could be observed. (**C**,**D**) Expression of *CA2* in the E120 cochlea. Broad expression of *CA2* in the spiral ligament fibrocytes could be detected at this stage. (**E**,**F**) Expression of *CA2* in the P0 cochlea. At this stage, *CA2* expression could be detected in type I, II, IV, and V spiral ligament fibrocytes. The nuclei were counterstained with Hoechst (blue). Scale bar 100 µm, *StV* Stria vascularis, *I* type I fibrocytes, *II* type II fibrocytes, *III* type III fibrocytes, *IV* type IV fibrocytes, *V* type V fibrocytes. (**A**–**F**) Basal turns.

**Figure 5:**
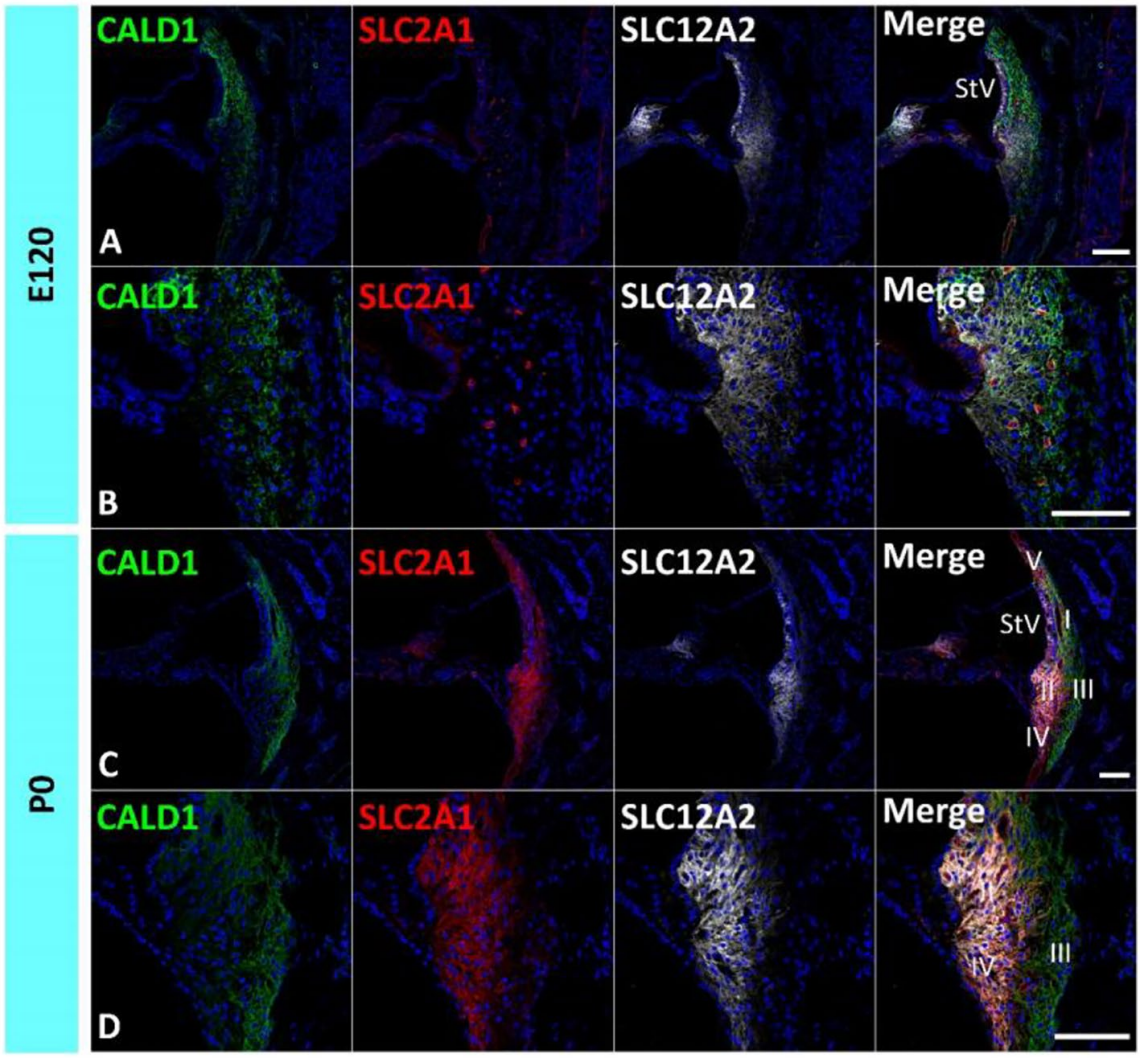
Expression of *SLC2A1*. (**A**,**B**) Expression of *SLC2A1* in the E120 cochlea. No *SLC2A1* expression could be detected in the spiral ligament fibrocytes, except for blood vessels. (**C**,**D**) Expression of *SLC2A1* in the P0 cochlea. At this stage, *SLC2A1* expression could be detected in type I, II, IV, and V spiral ligament fibrocytes. The nuclei were counterstained with Hoechst (blue). Scale bar 100 µm, *StV* Stria Vascularis, *I* type I fibrocytes, *II* type II fibrocytes, *III* type III fibrocytes, *IV* type IV fibrocytes, *V* type V fibrocytes. (**A**–**D**) Basal turns.

In cochlear spiral ligament fibrocytes, several characteristic expressions of the enzymes, such as *CA2*, *SLC2A1*, *AQP1*, and creatinine kinase are expressed, which are related to the regulation of endolymph homeostasis and transporters or channels to maintain normal hearing, are expressed^4^. In this study, we analyzed the developmental expression of *CA2* and *SLC2A1* (Figs. 4, 5).

*CA2* encodes carbonic anhydrase II, one of the 16 human α-carbonic anhydrase forms. *CA2* expression in the cochlea has been previously reported in rodents, humans, and common marmosets^16,26,37–41^. Carbonic anhydrase (CA) affects ion movement in spiral ligament fibrocytes by converting H_2_O and CO_2_ to H^+^ and HCO^-^ ions, which exchange for Na^+^, K^+^, and Cl^−^ and regulate the concentrations of these ions and pH in the cochlear fluid. Weak *CA2* expression was observed in the spiral ligament fibrocytes of a 15-week-old human fetus, whereas strong *CA2* expression was observed in the spiral limbus at this stage^41^.

In the developing cochlea of the common marmoset, *CA2* expression was not observed in spiral ligament fibrocytes by E115, whereas it was detected in the stria vascularis (Fig. 4A,B). At E120, *CA2* expression was detected in type I, II, IV, and V fibrocytes, similar to the expression pattern in the adult marmoset cochlea, as previously reported^16^ (Fig. 4C–F).

*SLC2A1* encodes glucose transporter 1 (GLUT1), which is present in the endothelial cells of the capillaries of the stria vascularis^7,42,43^. In the rodent cochlea, *SLC2A1* expression has also been observed in spiral ligament fibrocytes during late development^42^. In the developing cochlea of the common marmoset, *SLC2A1* expression was not observed in the spiral ligament by E120, whereas it was observed in several capillaries as previously reported^12^ (Fig. 5A,B). In the P0 cochlea, *SLC2A1* expression was detected in type I, II, IV, and V fibrocytes (Fig. 5C,D).

### Developmental expression patterns of type II collagen

**Figure 6:**
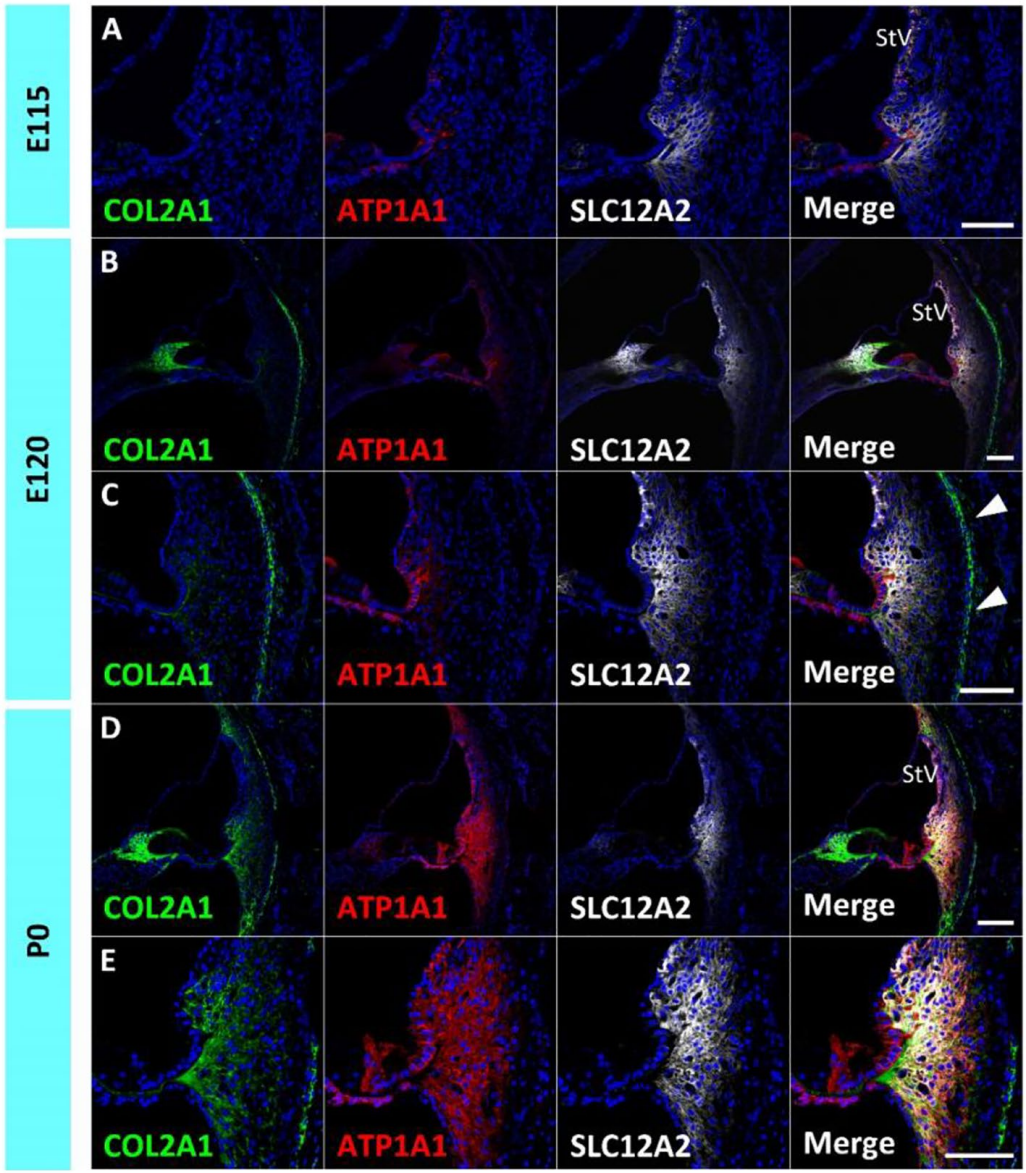
Expression of *COL2A1*. (**A**) Expression of *COL2A1* in the E115 cochlea. No *COL2A1* expression could be detected in the spiral ligament fibrocytes. (**B**,**C**) Expression of *COL2A1* in the E120 cochlea. At E120, *COL2A1* expression in the thin layer lining the cochlear boney capsule (arrowheads in **C**) and slight expression can be detected in fibrocytes. (**D**,**E**) Expression of *COL2A1* in the P0 cochlea. At P0 cochlear, abundant expression of the *COL2A1* is detected broadly in the lateral wall fibrocytes. The nuclei were counterstained with Hoechst (blue). Scale bar 100 µm, *StV* Stria Vascularis. (**A**–**E**) Basal turns.

Finally, this study examined the developmental expression patterns of type II collagen, the most abundant collagen in the cochlea and spiral ligament (Fig. 6). *COL2A1* encodes the alpha-1 chain of type II collagen, which is found in cartilage, the vitreous humor of the eye, and the inner ear. *COL2A1* is one of the causative genes for Stickler syndrome, which is characterized by ocular, skeletal, orofacial, and auditory defects^44^. *COL2A1* gene expression in spiral ligament fibrocytes in rodents^45^ and human fetuses^46^ has been reported previously.

In the developing cochlea of the common marmoset, no *COL2A1* expression was observed in spiral ligament fibrocytes at E115 (Fig. 6A). At E120, *COL2A1* expression was observed in the thin layer lining the cochlear bone capsule and its low expression was observed in fibrocytes (Fig. 6B,C). At P0, abundant expression of *COL2A1* was detected throughout the lateral wall fibrocytes (Fig. 6D,E).

## Discussion

In this study, we investigated the development of spiral ligament fibrocytes in common marmosets. We demonstrated the developmental expression patterns of the characteristic genes reported in rodents and non-human primates. Although spiral ligament fibrocytes are essential for normal hearing ability, little is known about their development compared to other cochlear tissues, such as hair cells, supporting cells, and spiral ganglion neurons. In addition, most of the existing developmental studies are based on rodent models, and only a handful of studies have investigated the spiral ligament fibrocytes development in human fetuses^7,41,46^.

Recently, interspecies differences in cochlear development between primates (including humans and common marmosets) and rodents have been reported. Therefore, studying the development of spiral ligament fibrocytes in humans is important. However, in the human fetus, spiral ligament fibrocyte development and differentiation occur in the second trimester of gestation (e.g., *COL2A1* expression in the human fetal cochlea is evident after 21–22 weeks of gestation^46^), making it more difficult to use due to ethical concerns in many countries recently. The common marmoset has been reported as an alternative research platform for human fetuses^12^ in the case of the development of the stria vascularis, an important developmental process that occurs in the late phase of gestation in humans^7^. In this study, we hypothesized that the common marmoset would be useful for studying the development of spiral ligament fibrocytes. We examined the gene expression patterns of developing spiral ligament fibrocytes.

**Figure 7:**
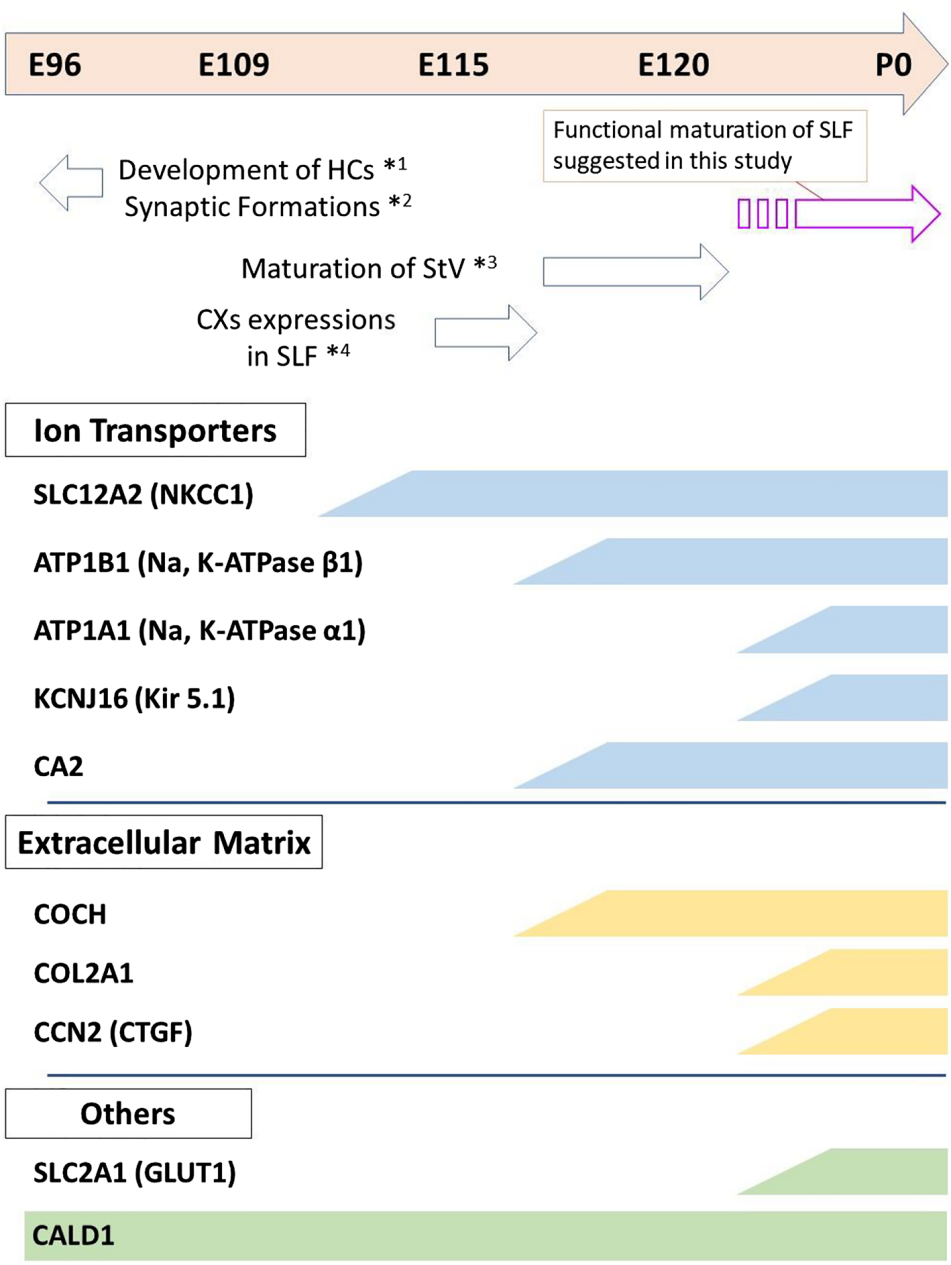
Schematic diagram of developmental gene expression patterns of spiral ligament fibrocytes. The expression of specific transporters became obvious after approximately E115. Expression of genes related to the ECM of spiral ligament fibrocytes is observed in relatively late stages of development, suggesting that functional differentiation precedes structural maturation of spiral ligament fibrocytes. *HCs* hair cells, *StV* stria vascularis, *CXs* Connexins, *SLF* spiral ligament fibrocytes. *1: Ref.^9^, *2: Ref.^10^, *3: Ref.^12^, *4: Ref.^49^.

Schematic diagrams of spiral ligament fibrocyte development in the common marmoset are shown in Fig. 7. Most of the expression of the characteristic genes and differentiation of spiral ligament fibrocytes, supported by the gene expression patterns, were detected in this primate at a relatively late stage of development, as predicted by previous observations obtained from in the human fetus^7^. Among the genes examined in this study, while *CALD1* expression was observed at the earliest stages, most of the initial expression of these characteristic markers, including transporters of channels required for essential functions of spiral ligament fibrocytes, was observed between E115 and P0. This indicates that the functional maturation of spiral ligament fibrocytes is evident during late phase of cochlear development in this primate. At the same time, characteristic markers for each subtype of spiral ligament fibrocytes appeared after E115. This indicates that the differentiation of these subtypes is evident during the late gestation phase in this primate. While the general developmental time course of the lateral fibrocytes of common marmosets was unveiled in this study, the tonotopic gradient of their development could not be investigated in detail. A future study will be awaited.

The developmental maturation of spiral ligament fibrocytes is essential for the regulation of high potassium concentrations in the endolymph and for the generation of sufficient endolymphatic potential for hearing ability. Endocochlear potential has previously been observed in rodents just prior to the onset of hearing and coincides with the morphological maturation of gaps and tight junctions^47,48^. If the emergence of an endocochlear potential in the common marmoset fetus remains unknown, we hypothesize that endocochlear potential and hearing do not emerge in the marmoset fetus before E115, based on the observation that tight junction formation in basal cells of the stria vascularis becomes evident and gap junctions in lateral wall fibrocytes mature after E115^12,49^. However, based on the expression patterns of important transporters, this study showed that spiral ligament fibrocytes are not fully mature at E115 (Fig. 7). Our results suggest that the predicted time of the endocochlear potential generation is later than at E120. Future studies measuring endocochlear potential in the fetuses of this primate may benefit from our speculation about the timing of endocochlear potential formation based on our histologic findings.

This study suggests a relatively long gap between the maturation of hair cells and spiral ganglion neurons (around E96)^9,10^ and the predicted time of emergence of the endolymphatic potential (after E120) in this primate (Fig. 7). During this period, synapse formation between the hair cells and spiral ganglion neurons and pruning of spiral ganglion neurons have been reported to occur^9,10^. How neuronal connections are refined in this primate without the sufficient endolymphatic potential to influence hair cell activity remains an important scientific question to be elucidated in the future. It might be also useful in this primate that examining Ca^2+^ channels or relating genes, which are essential for initial synaptic formations before sufficient endolymphatic potential formation, as previously reported in rodents^50^.

Our results revealed both interspecies similarities and interspecies differences between rodents and primates. Most of the sequential expression patterns of the genes examined in this study were conserved between rodents and primates; however, several interspecies differences should be noted. Among the transporters examined in this study, it has been previously reported that *Slc12a2* (P10 in gerbils^25^), *Atp1a1* (P10 in gerbils^26^, P10 in rats^27^), and *Ca2* (P8-12 in gerbils^26^, P5 in mice^37^) expression in spiral ligament fibrocytes precedes *Atp1b1* (P15 in gerbils^26^, P14 in rats^27^) and *Kcnj16* (P14 in rats^34^) expression in spiral ligament fibrocytes. In our observations of this primate, the sequential expression patterns between rodents and this primate were well preserved, with *SLC12A2* and *CA2* expressed relatively early and *KCNJ16* expressed in the late phase. However, we observed interspecies differences in the expression patterns of *ATP1A1* and *ATP1B1*; *ATP1B1* expression preceded *ATP1A1* expression in this primate, in contrast to rodents.

Genes related to the ECM and others were identified as having interspecies differences. Previous reports showed *COL2A1* expression in developing spiral ligament fibrocytes at P13 in mice^51^, *CCN2* expression at P0 in mice^24^, and *SLC2A1* expression at P14 in gerbils^42^. We have previously reported that the P0 mouse cochlea is histologically equivalent to E101 in the common marmoset, P9–E115, and P14–D0^9^. These results suggest that the expression patterns of *COL2A1* and *SLC2A1* are well conserved between rodents and this primate, whereas *CCN2* expression occurs relatively late in this primate. Expression patterns in humans have been reported for *COCH* and *COL2A1*; *COCH* expression in spiral ligament fibrocytes has been reported at 22 weeks of gestation^52^, and *COL2A1* at 21–22 weeks of gestation^46^. Furthermore, we have previously reported that the human fetal cochlea at 20 weeks of gestation is histologically equivalent to E115 in the common marmoset^9^. Thus, comparing previous findings with the current findings, we concluded that the timing of the expression patterns of *COCH* and *COL2A1* in humans and common marmosets is well conserved.

In this study, we revealed the developmental time course of spiral ligament fibrocytes in this primate model as an alternative to the human fetal cochlea. This understanding of spiral ligament fibrocytes in the primate will be useful in several ways. First, cochlear spiral ligament fibrocytes are targets for regenerative medicine following noise trauma^5,27,53^. Previous studies have shown that type IV spiral ligament fibrocytes are the most sensitive to noise and degenerate before hair cell loss^54,55^. In addition, changes in the expression of ion transporters, such as ATP1A1, in spiral ligament fibrocytes after noise trauma have been reported^56^. Therefore, this study would be useful for future regenerative therapy targeting primate spiral ligament fibrocytes, including in human patients.

Second, relationships between age-related hearing loss and degeneration or morphologic changes in spiral ligament fibrocytes have been reported in rodents^25,57,58^ and humans^59^. Recently, Sun et al. reported that the common marmoset is a useful model for age-related hearing loss in non-human primates^13^. Our findings of spiral ligament fibrocytes in this study would be useful for future studies focusing on the aging of spiral ligament fibrocytes in this primate.

Third, lateral wall fibrocytes are essential for regulating the immune system as well as ion homeostasis. In recent years, the importance of the immune system in the cochlea has been reported, particularly in acoustic trauma or drug-induced hearing loss^4^. Maturation or activation of the cochlear immune system occurs predominantly in the fibrocytes of the spiral ligament^5^. For example, intercellular adhesion molecule-1 (ICAM1) expression in the spiral ligament fibrocytes, which would be related to macrophage recruitment in the cochlea in these pathological models, has been reported^60–62^. In addition, Therefore, information on spiral ligament fibrocytes obtained in this study would also be useful for future studies on the development or maturation of the cochlear immune system in this primate.

## Conclusions

In conclusion, we have investigated the development of spiral ligament fibrocytes in the cochlea of a primate animal model. We have uncovered the underlying time course of spiral ligament fibrocyte development and the similarities and differences between these primates and rodents. The results of this study will be valuable for future developmental studies on the primate cochlea, as well as for studies on regenerative medicine or the aging studies of cochlear spiral ligament fibrocytes.

## Data Availability

All data generated or analyzed during this study are included in this published article.
